# The effect of sex and physical frailty on incident disability after 2 years among community-dwelling older adults: KFACS study

**DOI:** 10.1186/s12877-022-03263-5

**Published:** 2022-07-16

**Authors:** Seoyoon Lee, Miji Kim, Yunhwan Lee, Jinhee Kim, Hak Chul Jang, Belong Cho, Kyung Mook Choi, Eun Roh, Sang Joon Son, Jin-Hee Lee, Yong Soon Park, Sam-Gyu Lee, Bong Jo Kim, Hyeonju Kim, Chang Won Won

**Affiliations:** 1 Elderly Frailty Research Center, Department of Family Medicine, College of Medicine, Kyung Hee University, Seoul, Republic of Korea; 2 Interdisciplinary Graduate Program in Social Welfare Policy, Yonsei University, Seoul, Republic of Korea; 3 Department of Biomedical Science and Technology, College of Medicine, East-West Medical Research Institute, Kyung Hee University, Seoul, Republic of Korea; 4 Department of Preventive Medicine and Public Health, Ajou University School of Medicine, Suwon, Republic of Korea; 5 Department of Internal Medicine, Seoul National University Bundang Hospital, Seoul National University, College of Medicine, Seongnam, Korea; 6 Department of Family Medicine, Center for Health Promotion and Optimal Aging, Seoul National University College of Medicine & Hospital, Seoul, Republic of Korea; 7 Division of Endocrinology and Metabolism, Department of Internal Medicine, Korea University, Seoul, Republic of Korea; 8 Division of Endocrinology and Metabolism, Department of Internal Medicine, Hallym University Sacred Heart Hospital, Anyang, 14068 Republic of Korea; 9 Department of Psychiatry, Ajou University School of Medicine, Suwon, Republic of Korea; 10 Catholic institute of U-healthcare, The Catholic University of Korea, Seoul, Republic of Korea; 11 Department of Family Medicine, Hallym University Chuncheon Sacred Heart Hospital, Chuncheon, Republic of Korea; 12 Department of Physical & Rehabilitation Medicine, Chonnam National University Medical School, Gwangju, Republic of Korea; 13 Department of Psychiatry, College of Medicine, Gyeongsang National University, Jinju, Republic of Korea; 14 Department of Family Medicine, Jeju National University College of Medicine, Jeju, Republic of Korea; 15 Department of Family Medicine, Kyung Hee University Medical Center, Seoul, Republic of Korea

**Keywords:** Frailty, Disability incidence, Sex difference, Korea

## Abstract

**Background:**

This study investigated the impact of physical frailty on the development of disabilities in mobility, activities of daily living (ADL), and instrumental activities of daily living (IADL) according to sex among community-dwelling Korean older adults.

**Methods:**

We used data of 2,905 older adults aged 70-84 years from the Korean Frailty and Aging Cohort Study (KFACS) at baseline (2016-2017) and Wave 2 (2018-2019). Fried’s physical frailty phenotype was used to identify frailty.

**Results:**

After adjustment, frailty showed a higher impact for women than men on developing mobility disability (odds ratio [OR]=14.00, 95% confidence interval [CI]=4.8–40.78 vs. OR=9.89, 95% CI=4.28–22.86) and IADL disability after two years (OR=7.22, 95% CI=2.67–19.56 vs. OR=3.19, 95% CI=1.17–8.70). Pre-frailty led to mobility disability for women and men (OR=2.77, 95% CI=1.93–3.98 vs. OR=2.49, 95% CI=1.66–3.72, respectively), and IADL disability only for women (OR=3.01, 95% CI=1.28–7.09). Among the IADL components, both men and women who were prefrail or frail showed increased disability in ‘using transportation’. Among men, pre-frailty was significantly associated with disability in “going out” and “shopping”. In women, frailty was significantly associated with disability in “doing laundry,” “performing household chores,” “shopping,” and “managing money”.

**Conclusions:**

Physical frailty increased disability over 2 years for women more than men. Physical frailty increased disability in outdoor activity-related IADL components in men and household work-related IADL components in women. This study highlights the need for gender-specific policies and preventative programs for frailty, particularly restorative interventions that focus on women who are physically frail.

**Supplementary Information:**

The online version contains supplementary material available at 10.1186/s12877-022-03263-5.

## Background

According to the Pensions at a Glance (2019) report published by the Organization for Economic Co-operation and Development, Korea is expected to have 40% of its total population aged 65 years or older by 2050, making it the second oldest country in the world after Japan [1]. With the aging population, more attention has been paid to studies, policies, and programs to ensure the quality of life of older adults.

Old-age dependency and the cost of supporting a dependent aging population have increased recently in Korea [2]. The percentage of long-term care costs in national health insurance has risen to 12.27 percent by 2022, roughly doubling since 2008 [3, 4]. Moreover, older adults find it increasingly difficult to manage their daily lives alone, and the frequency with which they rely on others for help increases over time. Numerous studies have found that a decline in daily living activities is strongly linked to a low quality of life in old age [5–7]. To minimize dependency on others, it is important to prevent various physical and psychological problems associated with age, one of which is physical frailty.

Frailty refers to the age-related decline in physiological functions which weakens the body’s response to external stresses and increases the risk of disability and hospitalization [8]. The main clinical symptoms of frailty are reduced physical activity, decreased muscle mass, decreased energy, and decline in gait speed. Deterioration of these factors is associated with a cycle of frailty that progresses and worsens. Frailty is described as a dynamic transition from normal aging to pre-frailty to frailty and its complications [9, 10]. Therefore, it is of great importance to prevent this vicious cycle by actively changing behavior before the state of frailty is reached and minimizing risk factors in the pre-frail stage.

Numerous studies have shown an association between frailty and prospective development of disabilities worldwide [8, 11–16]. In Korea, one study was conducted in rural areas to investigate the association between frailty and disability in a cross-sectional manner [17]. However, none of the previously published prospective studies were conducted to comprehensively predict the development of activities of daily living (ADL), instrumental activities of daily living (IADL), and mobility limitations in association with frailty in old age among relatively healthy community-dwelling older adults in Korea nationwide.

Furthermore, it is well recognized that the prevalence and incidence of frailty and disability differs between men and women. Frailty is more common in women, and there are distinct differences in the prevalence of ADL, IADL, and mobility disability between men and women [5, 18–23]. Not only mere physical differences, such as biological or genetic factors but also differences in socioeconomic status or sociocultural experiences may have contributed to the differences in sex in health outcomes [24, 25]. From a socio-cultural perspective, men tend to play the role of supporting family primarily through employment based on formal relationships, whereas women are more likely to engage in informal and intimate interpersonal relationships and take care of the household. Those accumulated different experiences combined along with health behaviors may have resulted in different health outcomes for men and women [24, 26, 27].

Therefore, this study aimed to examine the effects of frailty and sex on the development of disability over two years. Based on the finding that the prevalence of frailty and disability differs between men and women, the impact of frailty on each item of the IADL may differ according to sex among Korean community-dwelling older adults.

## Methods

### Study population

**Fig. 1:**
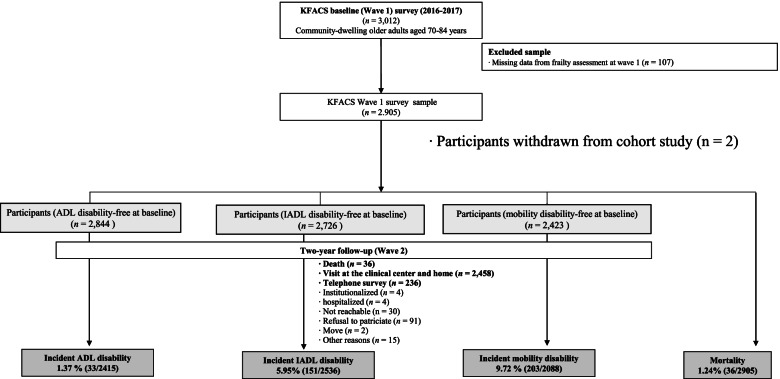
Data analysis flowchart

Data from the Korean Frailty and Aging Cohort Study (KFACS), the first cohort study on frailty in Korea, were used in this study. The KFACS recruited 3,014 community-dwelling older adults aged 70-84 years in 2016-2017 with a planned follow-up every 2 years. Participants in the KFACS were recruited from residents of urban and rural areas throughout the country at 10 study sites [28]. Further details can be found in the cohort profile report [29]. Of 3,014 participants at baseline, 2 withdrawn participants and 107 participants with missing data for frailty assessment were excluded; this study used data of 2,905 participants in baseline survey in 2016-2017 (Wave 1) and were followed up in 2018-2019 (Wave 2). At baseline, the participants were classified into three groups based on the absence of disability for the following criteria: ADL (*n* = 2,844), IADL (*n*=2,726), and mobility (*n*=1,444) (Fig. 1). At the 2-year follow-up 36 (1.24%) participants had died, 8 were admitted to long-term care facilities or hospitals, 32 were not reachable or moved out, and 91 refused to participate. A further 15 participants were excluded for other reasons (Fig. 1).

### Physical frailty assessment

Physical frailty was assessed using the Fried’s Frailty Phenotype, which consists of five components: unintentional weight loss, weakness, self-reported exhaustion, slowness, and low physical activity [8, 29, 30]. Scores ranged from 0 to 5, with 1 point allocated for each frailty component. Frailty status was determined by adding the scores for the five components: a score of 0 represents robust, 1–2 represents prefrailty, and 3 or more represents frailty.

### Outcomes (Disability and mortality)

This study investigated three types of disability: mobility, ADL, and IADL. For mobility disability, participants were asked whether they found it difficult to walk around the perimeter of a playground (approximately 400 m) or to climb a flight of stairs (10 steps) [31]. Participants who answered "very difficult" or "cannot do at all" to either of the questions were classified as having mobility disability. The Korean Activities of Daily Living and Korean Instrumental Activities of Daily Living scales were used to measure ADL and IADL disability [32]. The participants who answered ‘partially dependent’ or ‘fully dependent’ on any of the five ADL components (dressing, bathing, eating, transferring, and toileting) were classified as having an ADL disability.

IADL disability was also classified as part of the responses from those who responded 'partially dependent' or 'fully dependent' on more than two of the ten IADL components (grooming, performing household chores, cooking, doing laundry, going out, using transportation, shopping, managing money, using the telephone, and taking medication). Results from Wave 2 participants who responded by visiting one of the 10 centers, attending the clinic or receiving a home visit (*n*=2,458) were used to measure disability limitations in mobility, IADLs, and ADLs, and results from participants who responded only by attending the clinic, making home visits, and answering a telephone survey (*n*=236) were used to measure disability in IADLs. The cause and time of death during the follow-up survey were determined by interviewing family members or reviewing medical records (Fig. 1 and Supplementary Table S1). 

### Covariates

Sociodemographic factors, such as age, sex, educational level, area of residence, marital status, and economic status, including basic social security recipients and/or medical care aid recipients, were examined from the analysis. Smoking status, alcohol consumption, body mass index (BMI), and number of comorbidities were also included. Morbidity was defined as self-reported physician-diagnosed chronic disease (hypertension, myocardial infarction, dyslipidemia, diabetes mellitus, congestive heart failure, angina pectoris, peripheral vascular disease, cerebrovascular disease, osteoarthritis, rheumatoid arthritis, asthma, and chronic obstructive pulmonary disease).

### Statistical analysis

All analyses were performed using SPSS (version 25.0; SPSS Inc., Chicago, IL, USA) to examine differences among the robust, pre-frail, and frail groups. Analysis of variance and χ^2^ tests were performed. In addition, to assess the impact of frailty status on each disability and each item of IADL, multivariable logistic regression analysis was conducted, controlling for all covariates, and stratified by sex.

## Results

### Characteristics of the participants

The general characteristics of the participants according to their frailty status at baseline are shown in Table 1. Among the 2,905 participants, 277 (7.8 %) were frail, 1,366 were pre-frail (47.0 %), and 1,312 (45.2 %) were robust. The mean age of the frail participants was 78.6 years and 46.7% were women. The proportion of those with a low educational level in the frail group (67.8%) was significantly higher than that in the robust or prefrail groups. The proportion of patients receiving basic security and/or medical care was significantly higher in the frail (10.8%) group than in the robust (5.6%) and prefrail (7.8%) groups. Frail older adults had a higher number of comorbidities (2.1 ± 1.5) than the other groups. In terms of disabilities, frail older adults showed a significantly higher tendency to have mobility disabilities, ADL disabilities, and IADL disabilities (86.8%, 10.1%, and 17.6%, respectively) than the other groups (robust and prefrail).

**Table 1:** Baseline characteristics according to frailty status

Variable	Overall (*n* = 2905)	Robust (*n* = 1312)	Pre-frail (*n* = 1366)	Frail (*n* = 277)	*p-v*alue
Age (years)	76.0 ± 3.9	75.1 ± 3.6	76.4 ± 4.0	78.6 ± 3.6	<.001
70–74	1153 (39.7)	327 (47.8)	493 (36.1)	33 (14.5)	<.001
75–79	1080 (37.2)	500 (38.1)	492 (36.0)	88 (38.8)	
80–84	672 (23.1)	158 (14.1)	381 (27.9)	106 (46.7)	
Women	1524 (52.5)	618 (47.1)	777 (56.9)	129 (56.8)	<.001
Low education level (< 7 years)	1264 (43.5)	425 (32.4)	685 (50.1)	154 (67.8)	<.001
No spouse	948 (32.7)	363 (27.7)	490 (36.0)	94 (41.4)	<.001
Residence					
Urban	821 (28.4)	443 (34.0)	346 (25.4)	32 (14.2)	<.001
Suburban	1250 (43.3)	562 (43.1)	592 (43.5)	96 (42.5)	
Rural	818 (28.3)	298 (22.9)	422 (31.0)	98 (43.4)	
Basic livelihood security and/or medical care aid recipient^a^	204 (7.1)	74 (5.6)	106 (7.8)	24 (10.8)	.007
Current smoker	166 (5.7)	68 (5.2)	77 (5.6)	21 (9.3)	.050
Alcohol intake (≥ 2-3 time/week)	518 (17.8)	267 (20.4)	213 (15.6)	38 (16.7)	.005
Body mass index (kg/m^2^)	24.5 ± 3.0	24.4 ± 2.8	24.6 ± 3.1	24.3 ± 3.7	.256
Number of comorbidities	1.8 ± 1.3	1.5 ± 1.2	1.9 ± 1.3	2.1 ± 1.5	<.001
Comorbidities (≥2 among 13 diseases)	1578 (54.3)	622 (47.4)	816 (59.7)	138 (60.8)	<.001
Mobility disability	1396 (48.1)	390 (29.7)	809 (59.4)	197 (86.8)	<.001
ADL disability (≥1 point)	61 (2.1)	11 (0.8)	27 (2.0)	23 (10.1)	<.001
IADL disability (≥2 points)	179 (6.2)	51 (3.9)	88 (6.4)	40 (17.6)	<.001

*Notes*: Values are mean ± SD or number (%). Mobility disability: Participants who answered ‘difficult’ to walk around the perimeter of a playground (approximately 400 m) or to climb a flight of stairs (10 steps); ADL basic five activities of daily living (dressing, bathing, toileting, transferring, and feeding); IADL instrumental activities of daily living (grooming, performing household chores, cooking, doing laundry, going out, using transportation, shopping, managing money, using telephone, and taking medication); Number of comorbidities (self-reported physician-diagnosis of hypertension, myocardial infarction, dyslipidemia, diabetes mellitus, congestive heart failure, angina pectoris, peripheral vascular disease, cerebrovascular disease, osteoarthritis, rheumatoid arthritis, asthma, and chronic obstructive pulmonary disease). ^a^some missing data

Of the 2,844 participants who had no ADL limitation at baseline, 33 (1.37%) reported having ADL disability at 2-year follow-up. Of the 2,726 participants who had no limitations in IADLs at baseline, 170 (6.64%) reported difficulty in performing IADLs. Among the 1,444 subjects who had no mobility disability, 308 (23.43%) reported difficulty in mobility at the 2-year follow-up survey (Fig. 1).

### Mobility, ADL, and IADL disability prevalence at baseline and incidence after 2 years

Table 2 shows the prevalence of three disabilities (mobility, ADL and IADL), by sex at baseline, and the incidence of disability after 2 years. We have used the term prevalence to describe the percentage of disability at baseline, and incidence describe the percentage of disability after 2 years in older adults with no disability at baseline. For mobility and ADL disability, women showed a significantly higher prevalence and incidence of disability than men. Specifically, ‘dressing’ and ‘bathing’ showed a significant difference in prevalence at baseline between sex among the five items of the ADL scale. Only bathing showed a significant difference between men and women after two years (*p*=.008). There were also significant sex differences for IADL disability, with men having a statistically higher prevalence than women (*p*=.021), although there was no significant difference in incidence after two years between sexes. There was a higher prevalence of disability regarding household work – performing household chores (*p*=.007), cooking (*p*<.001), and doing laundry (*p*<.001) – for men, while women showed a higher prevalence for disability in using transportation (*p*<.001), managing money (*p*=.002), and using a telephone (*p*=.001). After two years, men had a significantly greater incidence of disability than women in performing home tasks (*p*=.017), cooking (*p*<.001) and taking medication (*p*=.008), than women.

**Table 2:** The prevalence of mobility, ADL, and IADL disability at baseline and their incidence after two years

Disability	Prevalence at baseline				Incidence after two years			
	Total (*n*=3010)	Men (*n*=1430)	Women (*n*=1580)	*p*-value	Total (*n*=1340)	Men (*n*=797)	Women (*n*=543)	*p*-value
**Mobility disability**	1396(48.1)	492 (35.7)	904 (59.4)	**<.001**	308(20.5)	134(17.2)	174(32.5)	**<.001**
Walk around 400 m	900(31.0)	283(20.5)	617(40.5)	**<.001**	127(9.7)	54(6.9)	73(13.6)	**<.001**
Climb 10 steps or stairs	1274(43.9)	433(33.9)	843(55.3)	**<.001**	281(21.4)	118(15.2)	164(30.4)	**<.001**
	Total (*n*=3012)	Men (*n*=1430)	Women (*n*=1582)	*p*-value	Total (*n*=2493)	Men (*n*=1187)	Women (*n*=1306)	*p*-value
**ADL disability**	61(2.1)	21(1.5)	40(2.6)	**.038**	33(1.4)	9(0.8)	24(1.9)	**.019**
Dressing	11(0.4)	9(0.7)	2(0.2)	**.023**	5(0.2)	3(0.3)	2(0.2)	.576
Bathing	54(1.9)	18(1.3)	36(2.4)	**.035**	30(1.2)	7(0.6)	23(1.8)	**.008**
Feeding	10(0.3)	7(0.5)	3(0.2)	.154	3(0.1)	2(0.2)	1(0.1)	.507
Transferring	4(0.1)	3(0.2)	1(0.1)	.271	3(0.1)	2(0.2)	1(0.1)	.507
Toileting	1(0.1)	1(0.1)	0(0.0)	.293	2(0.1)	1(0.1)	1(0.1)	.944
	Total (*n*=3012)	Men (*n*=1430)	Women (*n*=1582)	*p*-value	Total (*n*=2644)	Men (*n*=1245)	Women (*n*=1399)	*p*-value
**IADL disability**	179(6.2)	100(7.2)	79(5.7)	**.021**	170(6.6)	91(7.6)	79(5.8)	.074
Grooming	8 (0.3)	6(0.4)	2(0.1)	.119	25(1.0)	14(1.2)	11(0.8)	.360
Performing household chores	123(4.3)	73(5.3)	50(3.3)	**.007**	151(5.9)	85(7.1)	66(4.9)	**.017**
Cooking	168(5.8)	138(10.0)	30(2.0)	**<.001**	176(6.9)	128(10.7)	48(3.5)	**<.001**
Doing laundry	87(3.1)	62(4.5)	25(1.6)	**<.001**	91(3.6)	50(4.2)	41(3.0)	.118
Going out	24(1.0)	12(0.9)	12(0.8)	.808	52(2.0)	30(2.5)	22(1.6)	.116
Using Transportation^a^	87(3.0)	24(1.7)	63(4.1)	**<.001**	90(3.5)	38(3.2)	52(3.8)	.364
Shopping	53(2.1)	25(1.8)	28(1.8)	.957	84(3.3)	38(3.2)	46(3.4)	.754
Managing money	206(7.1)	77(5.6)	129(8.5)	**.002**	234(9.1)	102(8.5)	132(9.7)	.282
Using telephone	40(1.4)	9(0.7)	31(2.0)	**.001**	40(1.6)	20(1.7)	20(1.5)	.693
Taking medication^a^	15(0.5)	5(0.4)	10(0.7)	.268	37(1.4)	26(2.2)	11(0.8)	**.004**

*Notes*: Values are n (%); Prevalence = prevalence of disability at baseline; Incidence = the percentage of older adults with no disability at baseline who reported disability in Wave 2; Mobility disability: Participants who answered ‘difficult’ to walk around the perimeter of a playground (approximately 400 m) or to climb a flight of stairs (10 steps); Each ADL and IADL disability: number and percentage of the participants who answered as ‘partially dependent’ or ‘fully dependent’; *P*-value: chi-square *p*-value between incidence of men and women; ^a^some missing data

### Impact of frailty status on the incidence of disability in mobility, ADL, and IADL

Table 3 presents the impact of frailty status on the incidence of disability in mobility, ADL, and IADL by sex at two-year follow-up using univariable and multivariable logistic regression analyses. Supplementary Table S1 demonstrates the impact of frailty on the incidence of disability and mortality in all participants.

**Table 3:** Impact of frailty status on incident disability after 2 years

Incident disability	Frailty status at baseline	Men				Frailty status at baseline	Women			
		Univariable		Multivariable			Univariable		Multivariable	
		OR	95% CI	OR	95% CI		OR	95% CI	OR	95% CI
Mobility disability	Robust (*n* = 615)	*Ref*		*Ref*		Robust (n = 533)	*Ref*		*Ref*	
	Pre-frail (*n* = 482)	**2.60**	(1.99, 3.41)	**2.49**	(1.66,3.72)	Pre-frail (n = 659)	**2.95**	(2.33,3.75)	**2.77**	(1.93, 3.98)
	Frail (*n* =65)	**17.67**	(9.16, 34.08)	**9.89**	(4.28, 22.86)	Frail (n = 101)	**18.81**	(8.56,41.32)	**14.00**	(4.8, 40.78)
ADL disability	Robust (*n* = 611)	*Ref*		*Ref*		Robust (*n* = 524)	*Ref*		*Ref*	
	Pre-frail (*n* = 473)	6.46	(0.75,55.47)	0.00	(0.00, 0.00)	Pre-frail (*n* = 636)	2.27	(0.72,7.16)	0.78	(0.12, 5.17)
	Frail (*n* = 55)	**33.33**	(3.41,325.81)	0.00	(0.00, 0.00)	Frail (*n* = 83)	**14.20**	(4.28,47.18)	**10.71**	(1.21, 94.63)
IADL disability	Robust (*n* = 631)	*Ref*		*Ref*		Robust (*n* = 569)	*Ref*		*Ref*	
	Pre-frail (*n* = 486)	**2.18**	(1.4,3.38)	1.58	(0.81,3.08)	Pre-frail (*n* = 665)	**3.97**	(2.21,7.15)	**3.01**	(1.28,7.09)
	Frail (*n* = 59)	**7.55**	(4.2,13.57)	**3.19**	(1.17,8.70)	Frail (*n* = 86)	**16.07**	(8.28,31.16)	**7.22**	(2.67,19.56)

Multivariable: adjusted for age, low education level, marital status, residence, social security recipient, smoking status, alcohol intake, body mass index, number of comorbidities; Mobility disability: Participants who answered ‘difficult’ to walk around the perimeter of a playground (approximately 400 m) or to climb a flight of stairs (10 steps); ADL disability: ‘partially dependent’ or ‘fully dependent’ to any of five ADL components; IADL disability: ‘partially dependent’ or ‘fully dependent’ to two of ten IADL components

Univariable analysis showed that after 2 years, pre-frailty (odds ratio [OR]=2.60, 95% confidence interval [CI]=1.99–3.41 vs. OR=2.95, 95% CI=2.33–3.75) and frailty (OR=17.67, 95% CI=9.16–34.08 vs. OR=18.81, 95% CI=8.56–41.32) were associated with mobility disability for both men and women. After adjustment for covariates, frailty had a greater impact on developing mobility disability for women than men for pre-frail (OR=2.77, 95% CI=1.93–3.98 vs. OR=2.49, 95% CI=1.66–3.72, respectively) and frail participants (OR=14.00, 95% CI= 4.8–40.78 vs. OR=9.89, 95% CI=4.28–22.86, respectively). For the incidence of ADL disability, statistical significance was only reached for frail older men (OR=33.33, 95% CI=3.41–325.81) and women (OR=14.20, 95% CI=4.28–47.18), indicating that frailty has a greater influence on men in univariable analysis. After adjustment, the incidence of ADL was statistically significant for frail women (OR =10.71, 95% CI=1.21–94.63), while the influence of frailty on men was not evaluated owing to the small sample size.

In both men and women, IADL disability increased as frailty progressed, and the incidence of IADL disability was more obvious in women than in men in both univariable analysis and after adjustment. Before adjustment, both pre-frailty (OR=2.18, 95% CI=1.4–3.38) and frailty (OR=7.55, 95% CI=4.2–13.57) were significantly associated with IADL disability for men, but only the frail group (OR=3.19, 95% CI=1.17–8.70) showed an increased incidence of IADL disability after adjustment. As for women, in univariable and multivariable analyses, participants who were prefrail (OR=3.97, 95% CI=2.21–7.15 vs. OR=3.01, 95% CI=1.28–7.09) or frail (OR=16.07, 95% CI=8.28–31.16 vs. OR=7.22, 95% CI=2.67–19.56) at baseline had a higher risk of developing IADL disability.

### The impact of frailty on incident disability in specific-IADL items by sex

The impact of frailty on the incidence of the ten items of IADL disability by sex at two-year follow-up using univariable and multivariable logistic regression analysis is shown in Table 4. The impact of frailty on the incidence of each item of IADL disability in all participants is shown in Supplementary Table S2.

**Table 4:** Impact of frailty status on each item of IADL disability incidence by sex

Item	Frailty status at baseline	Men				Frailty status at baseline	Women			
		Univariable		Multivariable			Univariable		Multivariable	
		OR	95% CI	OR	95% CI		OR	95% CI	OR	95% CI
Grooming	Robust (*n* = 628)	*Ref*		*Ref*		Robust (n = 571)	*Ref*		*Ref*	
	Pre-frail (*n* = 492)	**14.04**	(1.81,109.12)	1.98	(0.15,25.51)	Pre-frail (n = 682)	5.86	(0.72,47.78)	2.91	(0.32,26.40)
	Frail (*n* =67)	**18.75**	(1.68,209.48)	0.00	(0, 0.00)	Frail (n = 95)	**18.03**	(1.86,175.16)	7.75	(0.61,99.24)
Performing household chores	Robust (*n* = 629)	*Ref*		*Ref*		Robust (*n* = 572)	*Ref*		*Ref*	
	Pre-frail (*n* = 503)	1.38	(0.87,2.21)	1.14	(0.55,2.37)	Pre-frail (*n* = 689)	**2.26**	(1.21,4.23)	2.02	(0.82,4.96)
	Frail (*n* = 69)	**2.79**	(1.32,5.91)	1.70	(0.51,5.67)	Frail (*n* = 98)	**7.20**	(3.36,15.46)	**4.99**	(1.57,15.82)
Cooking	Robust (*n* = 629)	*Ref*		*Ref*		Robust (*n* = 572)	*Ref*		*Ref*	
	Pre-frail (*n* = 503)	1.26	(0.86,1.84)	0.94	(0.51,1.74)	Pre-frail (*n* = 689)	**3.55**	(1.54,8.16)	1.90	(0.66,5.42)
	Frail (*n* = 69)	1.42	(0.67,3.01)	1.17	(0.36,3.74)	Frail (*n* = 98)	**11.26**	(4.32,29.40)	2.86	(0.75,10.88)
Doing laundry	Robust (*n* = 629)	*Ref*		*Ref*		Robust (*n* = 572)	*Ref*		*Ref*	
	Pre-frail (*n* = 503)	1.08	(0.58,2.02)	0.55	(0.19,1.55)	Pre-frail (*n* = 689)	**8.33**	(2.53,27.50)	**4.79**	(1.05,21.95)
	Frail (*n* = 69)	**4.14**	(1.82,9.39)	2.84	(0.74,10.93)	Frail (*n* = 98)	**19.18**	(5.09,72.20)	**9.92**	(1.77,55.50)
Going out	Robust (*n* = 629)	*Ref*		*Ref*		Robust (*n* = 572)	*Ref*		*Ref*	
	Pre-frail (*n* = 503)	**14.34**	(3.36,61.27)	**9.29**	(1.14,75.60)	Pre-frail (*n* = 689)	**13.58**	(1.80–102.68)	1.9.E+07	(0, 0.00)
	Frail (*n* = 69)	**29.86**	(5.9,151.04)	5.39	(0.31,94.38)	Frail (*n* = 98)	**30.70**	(3.55–265.71)	8.8.E+07	(0, 0.00)
Using transportation	Robust (*n* = 629)	*Ref*		*Ref*		Robust (*n* = 572)	*Ref*		*Ref*	
	Pre-frail (*n* = 503)	**8.52**	(2.95,24.57)	**12.70**	(1.59,101.12)	Pre-frail (*n* = 689)	**7.14**	(2.52,20.29)	**9.87**	(1.27,76.71)
	Frail (*n* = 69)	**20.49**	(6,70.02)	**21.49**	(2.03,227.65)	Frail (*n* = 98)	**25.66**	(8.32,79.18)	**28.50**	(3.15,257.65)
Shopping	Robust (*n* = 629)	*Ref*		*Ref*		Robust (*n* = 572)	*Ref*		*Ref*	
	Pre-frail (*n* = 503)	**6.80**	(2.59,17.85)	**5.86**	(1.25,27.42)	Pre-frail (*n* = 689)	**4.00**	(1.64,9.72)	4.29	(0.93,19.76)
	Frail (*n* = 69)	**14.09**	(4.34,45.72)	6.39	(0.81,50.49)	Frail (*n* = 98)	**13.16**	(4.81,35.99)	**9.85**	(1.67,57.90)
Managing money	Robust (*n* = 629)	*Ref*		*Ref*		Robust (*n* = 572)	*Ref*		*Ref*	
	Pre-frail (*n* = 503)	1.13	(0.74,1.74)	0.91	(0.48,1.73)	Pre-frail (*n* = 689)	**1.64**	(1.09,2.47)	**2.45**	(1.29,4.66)
	Frail (*n* = 69)	**2.30**	(1.13,4.66)	1.70	(0.58,5.02)	Frail (*n* = 97)	**4.12**	(2.31,7.35)	**2.87**	(1.15,7.12)
Using Telephone	Robust (*n* = 629)	*Ref*		*Ref*		Robust (*n* = 572)	*Ref*		*Ref*	
	Pre-frail (*n* = 503)	**3.49**	(1.11,11.04)	2.56	(0.25,25.90)	Pre-frail (*n* = 689)	**12.71**	(1.67,96.50)	6.27	(0.74,53.13)
	Frail (*n* = 69)	**12.21**	(3.2,46.61)	8.13	(0.6,110.91)	Frail (*n* = 98)	**24.30**	(2.69,219.76)	1.72	(0.09,31.73)
Taking medication	Robust (*n* = 629)	*Ref*		*Ref*		Robust (*n* = 572)	*Ref*		*Ref*	
	Pre-frail (*n* = 503)	**5.80**	(1.95,17.25)	4.88	(0.54,43.77)	Pre-frail (*n* = 689)	2.23	(0.59,8.45)	1.03	(0.18,6.05)
	Frail (*n* = 69)	**9.62**	(2.35,39.35)	8.58	(0.65,113.82)	Frail (*n* = 98)	0.00	(0,0.00)	0.00	(0,0.00)

Multivariable: adjusted for age, low education level, marital status, residence, social security recipient, smoking status, alcohol intake, body mass index, number of comorbidities. Bold text indicates significant ORs. *OR* odds ratio, *CI* confidence interval

After adjustment, for men, only the pre-frail group had an incidence of disability in the ‘going out’ (OR=9.29, 95% CI=1.14–75.60) and ‘shopping’ items (OR=5.86, 95% CI=1.25–27.42). Also for men, pre-frailty (OR=12.70, 95% CI=1.59–101.12) and frailty (OR=21.49, 95% CI=2.03–227.65) were both aligned with a significant increase in disability for ‘using transportation’. For women, compared to the robust group, frailty had the greatest impact on 'using transportation' for the pre-frail (OR=9.87, 95% CI=1.27–76.71) and frail groups (OR=28.50, 95% CI=3.15–257.65), and ‘doing laundry’ in both pre-frail and frail groups (OR=4.79, 95% CI=1.05–21.95; OR=9.92, 95% CI=1.77–55.50; respectively). Pre-frailty (OR=2.45, 95% CI=1.29–4.66) and frailty (OR=2.87, 95% CI=1.15–7.12) were both related to a substantial significant increase in the disability of ‘managing money’ compared to robust adults. Only the frail group had an incidence of disability of ‘shopping’ (OR=4.99, 95% CI=1.57–15.82) and ‘performing household chores’ (OR=9.85, 95% CI=1.67–57.90).

## Discussion

The main purpose of this study was to investigate the effects and sex differences of physical frailty on 2-year disability outcomes among community-dwelling older adults. Even after controlling for potentially confounding factors, physical frailty independently contributed to the incidence of disability in mobility and IADLs in both men and women. This result is consistent with the few previous studies in other countries that have investigated the incidence of disability in relation to physical frailty [11–15]. The impact of physical frailty on incident mobility disability was the strongest among the three disabilities investigated in both men and women in this study, while the impact of frailty on ADL disability in men was not measured due to the limited sample size. Furthermore, the impact of physical frailty on incident mobility and IADL disability was stronger and affected more items in women than in men (Table 3).

As shown in Table 2, women showed a higher prevalence of disability at baseline and incidence after two years than men. As shown in Table 3, the impact of both prefrailty and frailty on mobility disability was greater in women than in men. This result correlates with previous studies showing that the prevalence of mobility disability was higher in women than in men and increased exponentially with increasing age [12, 22, 23, 33]. In addition, women are more likely than men to progress from no disability to having a disability in climbing stairs and from intermittent to continuous disability [33]. Although the mobility assessment was based on self-reported data, previous research has demonstrated that self-reported and performance-based mobility measures for both men and women have a high level of concordance [34, 35]

Difficulty in bathing had the highest prevalence at baseline (1.9%) and incidence after 2 years (1.2%) among the ADL disability items assessed in Table 2. This finding is consistent with a prior study, which found that bathing is the first activity in which both older Americans and Chinese have difficulty [36]. The higher percentage of difficulty in bathing than other ADL items in this study also corresponds with the results of the 2017 Living Profiles of Older People Survey in Korea, which included 10,299 participants aged over 65 years – partially dependent on bathing (5.4%), dressing (2.5%), toileting (1.3), eating (1.1%), and transferring (0.7%), and fully dependent on bathing (1.5%), dressing (0.7%), toileting (0.7), eating (0.5%), and transferring (0.5%) [37].

As indicated in Table 3, the effect of frailty on IADL disability was also larger in women than in men after adjustment, whereas prefrailty had no statistically significant effect on IADL disability in men. Both prefrailty and frailty had the greatest impact on the 'using transportation' item for both men (OR=12.70; OR=21.49) and women (OR=9.87; OR=28.50), as shown in Table 4. It is reasonable to suppose that the item ‘using transportation’ is linked to mobility and that mobility disability adds to the construct of frailty, or vice versa, in both men and women.

It is also remarkable that physical frailty or prefrailty increased disability in outdoor activity-related items of IADL (going out, using transportation, and shopping) in men, while physical frailty was associated with disability in household work-related items (performing household chores, doing laundry) in older women. This can be interpreted as having an impact on the activities that the individual has been involved in, based on traditional gender roles. In previous study, Korean men said that they could not handle laundry, cook meals, or perform household chores, since these roles were unfamiliar to them [20].

Interestingly, frail older women had more difficulty in managing money than men. It is possible that older women lacked previous experience with similar tasks [20, 32]. This can also be explained by the higher risk of cognitive impairment in women than in men. According to prior studies, there are a number of factors related to cognitive function that differ according to age and sex, especially regarding working status and social participation [38, 39]. Working, using cell phones, using public transportation, and visiting a bank have traditionally been dependent on rigid gender roles in Korea. Because of physical and social factors, older women are more likely to require assistance with ‘cognitive tasks’ [20].

The substantial correlation between frailty and disability by sex may explain the disparity in frailty prevalence and average age: women made up 56.8% of the frail group, and their average age was higher than that of men, indicating that they were older and more frail (Table 1). According to a prior study comparing IADL disability by sex, women exhibited a higher tendency to report disabilities, use assistance, and a higher degree of disability than men [16, 20, 22, 23]. In contrast, Table 2 shows that the prevalence and incidence of IADL disability were higher in men than in women, particularly for the 'doing household chores,' 'cooking,' and 'doing laundry' components of the IADL item. Based on the data in Tables 3 and 4, by investigating the effect of frailty, it is reasonable to believe that physical frailty has a greater impact on women and that frailty aggravates household-related IADL disability in older women, whereas frailty worsens outdoor activity-related IADL disability in older men.

The prevalence of ADL and IADL disabilities (2.1% and 6.2%, respectively) at baseline according to Table 1 appears to be far lower for community-dwelling older adults. The low prevalence of ADL and IADL disabilities in this study sample at baseline may be attributed to the sampling method, which generally included ambulatory community-dwelling older adults. In contrast, the prevalence of ADL and IADL from the 2017 Living Profiles of Older People Survey in Korea was higher (8.7% and 16.6% respectively) [37].

As shown in Supplementary Table S1, the risk of developing ADL disability (OR=10.26) outweighed the risk of having IADL disability (OR=4.11) among frail participants after 2 years, whereas pre-frailty did not increase the risk of ADL disability in two years. Physical frailty was associated with increased mortality, similar to previous studies, whereas some studies showed conflicting results regarding mortality [11, 14]. Despite the different frailty measurements, the mortality results from this study were similar to those of a previous Korean study that utilized the frailty index to predict all-cause death in the Korean population based on age and sex [19].

Our study also has several limitations. The effect of sex differences on each ADL disability was not assessed in this study because of the small incidence of each item. In addition, the relatively low prevalence of each item in IADL may have influenced the results of this study. Furthermore, the frailty group has a small sample size and the gender was classified for the purpose of the analysis in this study, which might have had limited the statistical power. Finally, this study had a relatively short follow-up period (2 years) compared to other studies, which had a follow up of 4 to 11 years [11–13, 15].

Despite the limitations mentioned above, this study provides a better understanding of frailty that may help researchers and policymakers focus on frailty intervention programs in the Korean aging population, allowing them to provide more sophisticated interventions to prevent frailty. The increasing number of frail older adults is one of the biggest challenges facing health and social care. Frail older adults are vulnerable to developing disabilities, which lead to higher care needs and resource consumption. A higher prevalence of disability indicates a higher level of dependency, which ultimately leads to a higher need for support and a higher burden on family caregivers, the community, and the state. Therefore, efforts must be focused on managing frailty before it leads to an irreversible disability or other negative consequences. The authors found that the incidence of disability differed according to sex. To the best of our knowledge, this is the first study of its kind to investigate predicting the incidence of disability (mobility, ADL, and IADL) by sex difference using a nationwide sample collected by the KFACS team.

## Conclusion

In conclusion, this study found that physical frailty affects women more than men in terms of disability outcomes over 2 years. In women, frailty increased the risk of disability in mobility, ADL, and IADL, whereas in men, frailty increased the risk of disability in mobility and IADL. Physical frailty or pre-frailty increased disability in outdoor activity-related items of IADL (going out, using transportation, and shopping) in men, while physical frailty was associated with disability in household work (performing household chores, doing laundry) in older women. This study highlights the need for gender-specific policies and preventative programs for frailty, particularly restorative interventions that should focus on older women who are physically frail. Prospective studies are needed to analyze the prognosis of disability over time, with a longer follow-up period.

## Supplementary Information

**Additional file 1.** Supplementary Table

## Data Availability

The datasets used and/or analyzed during the current study are available from the corresponding author on reasonable request.

## Abbreviations

ADL: Activities of Daily Living
IADL: Instrumental Activities of Daily Living
KFACS: Korean Frailty and Aging Cohort Study
