# Supplementary material for: The effect of sex and physical frailty on incident disability after 2 years among community-dwelling older adults: KFACS study
Source: BMC Geriatr. 2022 Jul 16;22:588. doi: 10.1186/s12877-022-03263-5 (PMC9288076; doi:10.1186/s12877-022-03263-5)
Supplement: Supplementary file 1 — Additional file 1. Supplementary Table [file 12877_2022_3263_MOESM1_ESM.docx]

**Supplementary Table**

**Table S.1. Impact of frailty status on incident disability and mortality after 2 years**

| Incident disability and mortality | Frailty status  at baseline |  | Incidence  No. (%) | Univariate | |  | Multivariate | |
| --- | --- | --- | --- | --- | --- | --- | --- | --- |
|  |  |  |  | Odds Ratio | 95% CI |  | Odds Ratio | 95% CI |
| Mobility disability | *n* = 2088 |  |  |  |  |  |  |  |
|  | Robust (*n* = 1094) |  | 71 (6.5) | *Ref* |  |  | *Ref* |  |
|  | Pre-frail (*n* = 925) |  | 109 (11.8) | **1.93** | (1.41, 2.63) |  | **1.48** | (1.06, 2.05) |
|  | Frail (*n* = 69) |  | 23 (33.3) | **7.20** | (4.13, 12.55) |  | **5.11** | (2.78, 9.39) |
| ADL disability | *n* = 2415 |  |  |  |  |  |  |  |
|  | Robust (*n* = 1140) |  | 5 (0.4) | *Ref* |  |  | *Ref* |  |
|  | Pre-frail (*n* = 1125) |  | 16 (1.4) | **3.28** | (1.20, 8.97) |  | 2.34 | (0.83, 6.59) |
|  | Frail (*n* = 150) |  | 12 (8.0) | **19.74** | (6.85, 56.87) |  | **10.26** | (3.16, 33.31) |
| IADL disability | *n* = 2536 |  |  |  |  |  |  |  |
|  | Robust (*n* = 1197) |  | 43 (3.6) | *Ref* |  |  | *Ref* |  |
|  | Pre-frail (*n* = 1174) |  | 77 (6.6) | **1.88** | (1.29, 2.76) |  | **1.55** | (1.03, 2.32) |
|  | Frail (*n* = 165) |  | 31 (18.8) | **6.21** | (3.78, 10.19) |  | **4.11** | (2.35, 7.18) |
| Mortality | *n* = 2905 |  |  |  |  |  |  |  |
|  | Robust (*n* = 1312) |  | 10 (0.8) | *Ref* |  |  | *Ref* |  |
|  | Pre-frail (*n* = 1366) |  | 16 (1.2) | 1.54 | (0.70, 3.41) |  | 1.42 | (0.61, 3.28) |
|  | Frail (*n* = 227) |  | 10 (4.4) | **6.00** | (2.47, 14.59) |  | **4.40** | (1.56, 12.42) |
| Multivariate analysis adjusted for: age, sex, low education level, marital status, residence, social security recipient, smoking status, alcohol intake, body mass index, number of comorbidities; Mobility disability: participants who answered ‘difficult’ to walk around the perimeter of a playground (approximately 400 m) or to climb a flight of stairs (10 steps); ADL disability: ‘partially dependent’ or ‘fully dependent’ to any of five ADL components; IADL disability: ‘partially dependent’ or ‘fully dependent’ to two of ten IADL components. | | | | | | | | |

**Table**

**S.2. Impact of frailty status on the incidence of each item of IADL disability**

| Item | Frailty status  at baseline | Univariate | | Multivariate | |
| --- | --- | --- | --- | --- | --- |
|  |  | OR | 95% CI | OR | 95% CI |
| Grooming  (*n* = 2724) | Robust (*n* = 1248) | *Ref* |  | *Ref* |  |
|  | Pre-frail (*n* = 1273) | **9.94** | (2.32, 42.63) | 2.56 | (0.52, 12.66) |
|  | Frail (*n* = 203) | **42.63** | (9.54, 190.37) | **10.00** | (1.72, 58.19) |
|  |  |  |  |  |  |
| Performing household chores  (*n* = 2724) | Robust (*n* = 1248) | *Ref* |  | *Ref* |  |
|  | Pre-frail (*n* = 1273) | **1.80** | (1.28, 2.54) | 1.42 | (0.85, 2.36) |
|  | Frail (*n* = 203) | **6.66** | (4.35, 10.19) | **3.67** | (1.9, 7.10) |
|  |  |  |  |  |  |
| Cooking  (*n* = 2724) | Robust (*n* = 1248) | *Ref* |  | *Ref* |  |
|  | Pre-frail (*n* = 1273) | **1.46** | (1.07, 1.98) | 1.15 | (0.72, 1.86) |
|  | Frail (*n* = 203) | **3.31** | (2.14, 5.10) | **2.52** | (1.29, 4.93) |
|  |  |  |  |  |  |
| Doing laundry  (*n* = 2724) | Robust (*n* = 1248) | *Ref* |  | *Ref* |  |
|  | Pre-frail (*n* = 1273) | **2.04** | (1.3, 3.21) | 1.24 | (0.63, 2.42) |
|  | Frail (*n* = 203) | **9.06** | (5.41,15.17) | **4.86** | (2.19, 10.78) |
|  |  |  |  |  |  |
| Going out  (*n* = 2724) | Robust (*n* = 1248) | *Ref* |  | *Ref* |  |
|  | Pre-frail (*n* = 1273) | **10.87** | (3.89, 30.38) | **6.40** | (1.44, 28.49) |
|  | Frail (*n* = 203) | **43.68** | (15.03, 126.97) | **18.43** | (3.6, 94.20) |
|  |  |  |  |  |  |
| Using transportation  (*n* = 2595) | Robust (*n* = 1238) | *Ref* |  | *Ref* |  |
|  | Pre-frail (*n* = 1198) | **7.75** | (3.99, 15.06) | **8.82** | (2.67, 29.12) |
|  | Frail (*n* = 159) | **34.26** | (16.91, 69.42) | **23.72** | (6.58, 85.52) |
|  |  |  |  |  |  |
| Shopping  (*n* = 2601) | Robust (*n* = 1235) | *Ref* |  | *Ref* |  |
|  | Pre-frail (*n* = 1203) | **5.53** | (3.04, 10.04) | **4.83** | (1.84, 12.67) |
|  | Frail (*n* = 163) | **23.31** | (12.21, 44.51) | **12.18** | (4.11, 36.06) |
|  |  |  |  |  |  |
| Managing money  (*n* = 2723) | Robust (*n* = 1248) | *Ref* |  | *Ref* |  |
|  | Pre-frail (*n* = 1273) | **1.64** | (1.24, 2.17) | **1.72** | (1.14, 2.60) |
|  | Frail (*n* = 202) | **4.00** | (2.7, 5.91) | **2.51** | (1.37, 4.58) |
|  |  |  |  |  |  |
| Using Telephone  (*n* = 2663) | Robust (*n* = 1243) | *Ref* |  | *Ref* |  |
|  | Pre-frail (*n* = 1238) | **6.83** | (2.66, 17.51) | **5.09** | (1.15, 22.63) |
|  | Frail (*n* = 182) | **28.68** | (10.68, 77.01) | **9.30** | (1.83, 47.19) |
|  |  |  |  |  |  |
| Taking medication  (*n* = 2673) | Robust (*n* = 1241) | *Ref* |  | *Ref* |  |
|  | Pre-frail (*n* = 1243) | **4.14** | (1.81, 9.48) | 2.37 | (0.63, 8.96) |
|  | Frail (*n* = 189) | **13.13** | (5.23, 32.96) | **5.67** | (1.26, 25.53) |
| Multivariate analysis adjusted for: age, sex, low education level, marital status, residence, social security recipient, smoking status, alcohol intake, body mass index, number of comorbidities. | | | | | |
